# The Effects of Repeated Dyspnea Exposure on Response Inhibition

**DOI:** 10.3389/fphys.2019.00663

**Published:** 2019-05-28

**Authors:** Josef Sucec, Michaela Herzog, Omer Van den Bergh, Ilse Van Diest, Andreas von Leupoldt

**Affiliations:** Health Psychology, University of Leuven, Leuven, Belgium

**Keywords:** breathlessness, dyspnea, habituation, inhibition, LPC, N400

## Abstract

In order to treat dyspnea (=breathlessness) successfully, response inhibition (RI) as a major form of self-regulation is a premise. This is supported by research showing that self-regulation is associated with beneficial behavioral changes supporting treatment success in patients. Recent research showed that dyspnea has an impairing effect on RI, but the effects of repeated dyspnea exposure on RI remain unknown. Therefore, the present study tested the effects of repeated resistive load-induced dyspnea on RI over a 5-day period. Healthy volunteers (*n* = 34) performed the standard version of the Stroop task during baseline and dyspnea conditions on the first and fifth testing day and underwent an additional dyspnea exposure phase on each testing day. Variables of interest to investigate RI were reaction time, accuracy as well as the event-related potentials late positive complex (LPC) and N400 in the electroencephalogram. Reduced accuracy for incongruent compared to congruent stimuli during the dyspnea condition on the first testing day were found (*p* < 0.001). This was paralleled by a reduced LPC and an increased N400 for incongruent stimuli during the induction of dyspnea (*p* < 0.05). After undergoing dyspnea exposure, habituation of dyspnea intensity was evident. Importantly, on the fifth testing day, no differences between baseline, and dyspnea conditions were found for behavioral and electrophysiological measures of RI. These findings demonstrate that the impairing effect of dyspnea on RI disappeared after repeated dyspnea exposure in healthy participants. Translated to a clinical sample, it might cautiously be suggested that dyspnea exposure such as dyspnea perceived during physical exercise could reduce the impairing effect of dyspnea on RI which might have the potential to help increase self-regulation abilities and subsequent treatment efforts in dyspneic patients.

## Introduction

Dyspnea, also commonly known as breathlessness, is defined as the “subjective experience of breathing discomfort that consists of qualitatively distinct sensations that vary in intensity” (American Thoracic Society, 1999, p. 322). It is considered a multidimensional bodily sensation consisting of a distinguishable sensory (=intensity of dyspnea) and affective dimension (=unpleasantness of dyspnea) (American Thoracic Society, 1999; von Leupoldt et al., 2006; Lansing et al., 2009; Parshall et al., 2012; Wan et al., 2012; Sharma et al., 2016). Dyspnea is a major symptom in many disorders including respiratory, cardiovascular, neuromuscular, cancerogenic, and psychological disorders (Smoller et al., 1996; Solano et al., 2006). Furthermore, dyspnea is also a common symptom in healthy subjects during strenuous exercise, stressful and anxiety-provoking situations, and in high altitude (Gigliotti, 2010). Notably, dyspnea is a highly disabling symptom impairing performance and functioning in daily life including cognitive functioning (Currow et al., 2017; Johnson and Gozal, 2018; van Beers et al., 2018).

Repeated experiences of dyspnea can play an important role in shaping the perception of dyspnea. Previous research showed that repeated dyspnea exposure frequently results in reduced perception (Wadell et al., 2013; Donesky et al., 2014; Herigstad et al., 2017) as well as neural processing of dyspnea (habituation) (Von Leupoldt et al., 2011; Stoeckel et al., 2015). This was found for both the affective and sensory dimension of dyspnea (Stoeckel et al., 2015) while some research indicates stronger effects for the affective dyspnea dimension (Wan et al., 2009; Wadell et al., 2013; Donesky et al., 2014; Herigstad et al., 2017). However, laboratory research typically investigates repeated dyspnea exposure within a shorter time frame (i.e., within one testing session) (Wan et al., 2009; Von Leupoldt et al., 2011; Stoeckel et al., 2015). Longer time frames, which might more closely mirror clinical dyspnea experiences in everyday life, are commonly not explored. Notably, the effects of repeated dyspnea exposure are also affected by the characteristics of participants. For example, it was demonstrated that anxiety affects the response to repeated dyspnea exposure leading to less habituation or even increased dyspnea (sensitization) in high anxious compared to low anxious individuals (Wan et al., 2006, 2012). So far, potential effects of repeated dyspnea exposure on cognitive functioning have not been explored.

Impaired cognitive functioning in patient populations experiencing dyspnea such as patients with chronic obstructive pulmonary disease (COPD) or asthma has already been documented (Dodd et al., 2010; Cleutjens et al., 2014, 2018; Irani et al., 2017; van Beers et al., 2018). However, the impairing acute effect of a dyspneic stimulus on these cognitive functions has never been explored in patients. Notably, the relevance of dyspnea for cognitive functioning is supported by recent research in healthy subjects which showed that experimentally induced dyspnea impairs cognition in various cognitive tasks (Nierat et al., 2016; Sucec et al., 2018a; Vinckier et al., 2018; Sucec et al., unpublished). For example, Sucec et al. (2018b) found an impairing effect of experimentally induced dyspnea on behavioral and electrophysiological measures of response inhibition (RI) in healthy participants. RI itself can be defined as the inhibition of automatic, prepotent, or inappropriate responses (Miyake et al., 2000) and is a major form of self-regulation which is important for self-control of behavior, emotions, and thoughts (Baumeister, 2014). Research showed that increased self-regulation is associated with intended changes in lifestyle and reduced behavioral risk factors during or after rehabilitation in patients (Sniehotta et al., 2005) and a premise to induce behavioral changes that are targeted in many established treatment procedures including the treatment of dyspnea (Bourbeau et al., 2004; Celli et al., 2004). For example, conventional interventions in dyspneic patients such as quitting tobacco consumption, correct intake of medication, engaging in a more active lifestyle, and adaption to a healthier nutrition plan, all typically rely on appropriate self-regulation in order to achieve a level of self-control that allows inhibition of undesired habitual behavioral responses (e.g., refrain from tobacco consumption, overeating, extensive amount of time sitting in front of a TV). This is a premise to facilitate more behavioral responses that are in line with guidelines for dyspnea management (e.g., use of nicotine gum, stop eating when full, decide to take a walk) (American Thoracic Society, 1999; Celli et al., 2004). Since research suggests that dyspnea has an impairing effect on RI (Van Diest et al., 2000; Sucec et al., 2018b) it might be speculated that repeated dyspnea exposure could alter this effect via habituation. However, this has never been explored before. If repeated dyspnea exposure reduces the impairing effect of dyspnea on RI this could positively affect treatment outcomes and the course of the disease in patients suffering from dyspnea by increasing self-regulation abilities (Celli et al., 2004; Nici and ZuWallack, 2012; Baumeister, 2014).

Response inhibition is oftentimes investigated with the color-word Stroop task which is characterized by good to excellent test-retest reliability (Harbeson et al., 1982; MacLeod, 1991; Siegrist, 1997; Strauss, 2005). Furthermore, its’ external validity for behavioral self-control has already been established. For example, reduced inhibition during the performance of the color-word Stroop task is associated with the inability to refrain from smoking which is typically considered one of the most relevant dyspnea management strategies (Celli et al., 2004; Krönke et al., 2015; Yip et al., 2016). During the color-word Stroop task, four different words are presented either in congruent ink color and semantic meaning (e.g., “blue” presented in blue color) or in incongruent ink color and semantic meaning (e.g., “blue” presented in red color). Since the correct execution of the task demands the fast and correct indication of the ink color, participants need to inhibit the prepotent response to indicate the semantic meaning of the color-word solely during presentation of incongruent color-words (MacLeod, 1991; Badzakova-Trajkov et al., 2009). Consequently, incongruent words are typically processed slower resulting in slower reaction times which is widely known as the Stroop effect (Liotti et al., 2000; Miyake et al., 2000; Hanslmayr et al., 2008; Badzakova-Trajkov et al., 2009). Besides the application of behavioral measures (reaction time, accuracy), RI in the Stroop task is typically investigated with the technique of event-related potentials (ERPs) in the electroencephalogram (EEG). Typically, ERPs of interest include the late positive complex (LPC) and the N400 (Rebai et al., 1997; Hanslmayr et al., 2008; Badzakova-Trajkov et al., 2009).

We previously published the cross-sectional analysis (T1) of the current study elsewhere (Sucec et al., 2018b) in which we reported impaired RI during dyspnea indicated by reduced accuracy, and reduced LPC as well as increased N400 amplitudes for incongruent color words during experimentally induced dyspnea (for details: Sucec et al., 2018b). However, the potential effects of subsequent repeated dyspnea exposure remained unclear from our previous report. Therefore, the aim of the current study was to examine whether repeated dyspnea exposure over a 5-day interval reduces the initial impairing effect of dyspnea on RI indicated by behavioral and electrophysiological variables. The four color-word Stroop task (Stroop, 1935) was performed during a baseline and a resistive load-induced dyspnea condition on the first (T1) and fifth testing day (T5) while high-density EEG was continuously measured. During all five testing days, participants underwent an additional resistive load-induced dyspnea exposure phase. We hypothesized that dyspnea exposure over a 5-day period would reduce dyspnea perception. Furthermore, we expected that this habituation of dyspnea would result in less impairments in RI indicated by less impaired behavioral performance (faster reaction time, more accuracy) and more comparable electrophysiological responses (LPC, N400) during dyspnea relative to baseline conditions. Additionally, an explorative analysis examined the role of anxiety on dyspnea perception and RI over time.

## Materials and Methods

The cross-sectional in-depth analysis of the current study (T1) was previously published elsewhere (Sucec et al., 2018b). The current study contains the longitudinal analysis with an exclusive focus on habituation. Large parts of the methods were previously described in detail in Sucec et al. (2018b).

### Participants

Thirty-five healthy participants (25 females, age range: 18–41 years) underwent the current study on all five testing days and received either course credits or 40 € as compensation. Exclusion criteria included pregnancy, self-reported physiological/psychological disorders, consumption of alcohol, nicotine, and/or medication 24 h prior to testing on all five testing days. Normal baseline pulmonary function was confirmed via standard spirometry on the first and fifth testing day (Miller et al., 2005). The medical ethics committee of the hospital of the University of Leuven gave approval for this study (S57727).

### Anxiety

All participants filled out the Dutch version of the State-Trait Anxiety Inventory (STAI; Spielberger et al., 1970; Van der Ploeg, 1980) with 40 items in total. The STAI consists of two subscales each containing 20 items that measure levels of state (STAI-S) and trait anxiety (STAI-T). The STAI-S asks how participants feel right now with the scale ranging from 1 (“not at all”) to 4 (“very much so”) while the STAI-T asks how participants generally feel on a scale ranging from 1 (“almost never”) to 4 (“almost always”). Greater summary scores indicate higher levels of anxiety. The STAI showed good to excellent psychometric properties including test-retest reliability as well as construct and concurrent validity (Spielberger, 1989).

### Dyspnea Induction

Dyspnea was induced via breathing through inspiratory resistive loads (Hans Rudolph Inc., Shawnee, KS, United States) on all testing days (Brack et al., 1998; Webster and Colrain, 2000; Herzog et al., 2018a; Stoeckel et al., 2018). Prior to the experimental phase on T1 and T5, maximal inspiratory pressure (MIP) was measured with a handheld electronic device (POWERbreathe, HaB International Ltd, Southam, United Kingdom) to control for possible training effects with higher MIP values indicating greater muscular strength and capacity of the respiratory apparatus (Wijkstra et al., 1994). Furthermore, participants underwent a load magnitude estimation task (Herzog et al., 2018c, 2019; Sucec et al., 2018a) on T1 to pre-determine the individual inspiratory resistive load level that corresponds best with a dyspnea intensity rating of “strong” being represented by a “5” on a modified Borg scale (range: 0–10; Borg, 1990). This magnitude of dyspnea was used to induce strong dyspnea on all testing days. Finally, to keep the experimental protocol and the strain on the respiratory muscles between T1 and T5 identical for each participant the load estimation task was repeated on T5.

### Stroop Task

The standard version of the Stroop task (Stroop, 1935) was performed where the indication of the ink color of a color-word via button press on a response box was required. One of four color-words was either presented in its respective color (=congruent) or in a different color (=incongruent). All possible combinations were presented semi-randomly on a standard 32-inch monitor with the number of congruent and incongruent color-words being equal. Stimulus presentation was maximally 2 s or until the participant pressed a button of the response box, followed by an inter-trial interval of minimally 2 s with a fixation mark (+) being presented, resulting in a total trial duration of exactly 4 s.

All participants performed the Stroop task under both experimental conditions (baseline, dyspnea) on T1 and T5. Both conditions consisted of two blocks with all blocks being alternated and counterbalanced (either “Dyspnea-Baseline-Dyspnea-Baseline” or vice versa). Eighty trials per block were presented (=5 min 20 s) resulting in a total of 160 trials for each condition (=10 min 40 s) and 320 trials for the whole experiment (=21 min 20 s) on T1 as well as T5.

### Dyspnea Exposure Phase

The dyspnea exposure phase on the five testing days included three baseline and three dyspnea blocks each lasting for eight full breaths. All blocks were presented in alternating counterbalanced order. To induce dyspnea during the dyspnea blocks the predetermined inspiratory resistive load from the load magnitude estimation task of T1 was used. After each block ratings on the dyspnea dimensions intensity and unpleasantness were obtained. Importantly, the identical dyspnea exposure phase was repeated on all five testing days (T1 – T5).

### Ratings of Dyspnea and Affective State

Dyspnea was explained as “difficult and uncomfortable breathing.” To obtain dyspnea intensity and dyspnea unpleasantness ratings a visual analog scale (Aitken, 1969) was used. This scale ranged from 0 (for intensity: “not noticeable”; for unpleasantness: “not unpleasant”) to 100 (for intensity: “maximally imaginable intensity”; for unpleasantness: “maximally imaginable unpleasantness”). Additionally, the 9-point Self-Assessment Manikin (Bradley and Lang, 1994) was used for ratings of valence ranging from unpleasant to pleasant and arousal ranging from calm to aroused. All ratings referred to the preceding block and were obtained via mouse press, which was digitally stored.

### Psychophysiological Recordings and Data Reduction

**FIGURE 1:**
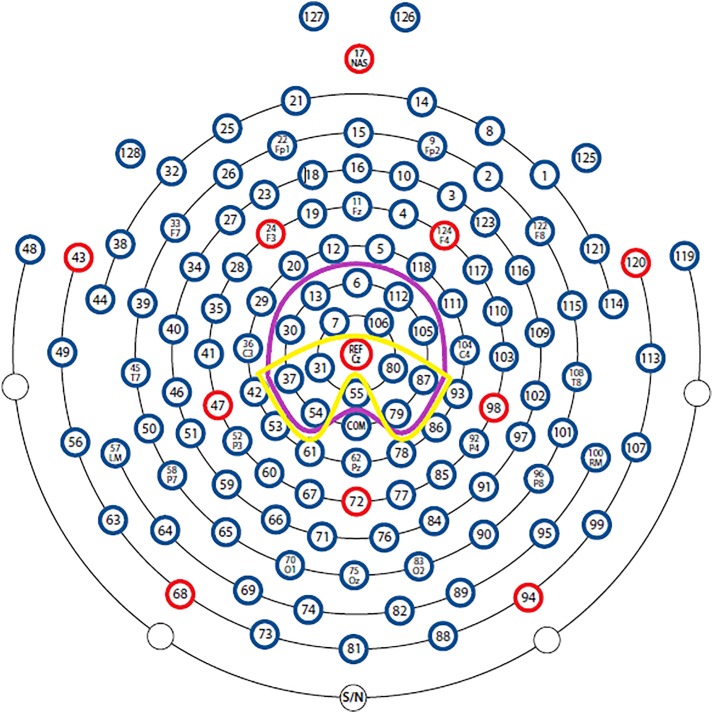
Sensor outline of the HydroCel Geodesic Sensor Net (with permission from Electrical Geodesics, Inc. Electrodes). The LPC and N400 were calculated with the sensors framed with yellow and purple color, respectively.

A 129 channel EEG system (Philips Electrical Geodesics Inc., Eugene, United States) sampling at 250 Hz with Cz as reference was used to record brain activity during each condition. Impedance level was kept below 50 kΩ throughout the experiment. EEG data were processed offline using BESA 6.0 (BESA GmbH, Gräfelfing, Germany). Filters (0.1 – 30 Hz, notch: 50 Hz) were applied to the raw data. An adaptive artifact correction (Ille et al., 2002) was used, and based on established recommendations (Luck, 2014) the data were re-referenced offline from Cz to the average reference. Stimulus-locked epochs of 1200 ms starting at -200 ms pre-stimulus were averaged. The first 100 ms before the stimulus were used as baseline. Based on research using the color-word Stroop task (West and Alain, 1999; Liotti et al., 2000) the LPC was identified as the mean between 400 and 600 ms after stimulus onset in centro-parietal areas. The N400 was identified as mean voltage ranging from 350 to 700 ms in centro-parietal areas (Figure 1). For electrode selection, visual inspection of the signal and official guidelines were used (Duncan et al., 2009).

Participants were respiring through a breathing circuit with the nose being occluded by a nose clip. The breathing circuit consisted of a mouthpiece connected to a two-way non-rebreathing t-shaped valve (Hans Rudolph Inc., Shawnee, KA, United States). On the expiratory side of the valve, PETCO_2_ was measured continuously with a capnograph (Nonin Medical Inc., Plymouth, MA, United States). Furthermore, mouth pressure was measured from the center of the valve. The inspiratory side of the valve was connected via tubing to a pneumotachograph which measured airflow. Subsequently, a loading manifold (all Hans Rudolph Inc., Shawnee, KA, United States) was connected through which the individually calibrated resistive loads were introduced. The continuously recorded PETCO_2_, mouth pressure, and airflow were used to calculate the respiratory variables breathing frequency (f), inspiratory time (T_I_), tidal volume (V_T_), mean airflow (V’), peak inspiratory mouth pressure (P_Imax_), and PETCO_2_ with AcqKnowledge 4.2 (Biopac, Goleta, CA, United States).

### Procedure

At the beginning of T1, participants filled out the informed consent form. The following protocol was identical for T1 and T5: Firstly, participants performed a standard spirometry, followed by the measurement of the MIP. Prior to the experimental phase, participants filled out the STAI-S and STAI-T and received detailed instructions. Then the load magnitude estimation task followed. Afterward, the EEG net was mounted and a dyspnea exposure phase followed. Subsequently, the Stroop task was explained followed by 40 practice trials. Unless the accuracy was higher or equal to 80 percent the practice trials were repeated. In our sample maximally one repetition of the practice phase was needed. Then the actual experimental phase followed. Throughout this phase, participants underwent the four blocks of the Stroop task which were performed under both experimental conditions. During all conditions, participants were instructed to breathe as normally as possible. Ratings of dyspnea and affective state were obtained after each block. This was followed by a short break. Finally, if applicable a reminder for the upcoming session was given.

### Statistical Analysis

From the cross-sectional analysis (T1) where we excluded two participants due to excessive noise in the EEG (for details: Sucec et al., 2018b), additionally three participants were excluded, two because of excessive artifacts (i.e., muscle/movement artifacts) in the EEG during T5 and one participant was not able to participate after T3. The final longitudinal analysis included 31 participants (Table 1). Data were analyzed with SPSS 24. Averages were calculated for the two blocks of each experimental condition and of the three blocks of each dyspnea exposure phase. A 2 × 2 × 2 repeated measure analyses of variance (ANOVA) with the within factors condition (baseline, dyspnea), congruency (congruent, incongruent), and time (T1, T5) was calculated for reaction times, accuracy, the LPC, and the N400. Multiple 2 × 2 repeated measure ANOVAs with the within factors condition (baseline, dyspnea) and time (T1, T5) were calculated for ratings of dyspnea and affective state as well as respiratory variables. Moreover, 2 × 5 repeated measure ANOVAs with the within factors condition (baseline, dyspnea) and time (T1, T2, T3, T4, and T5) were calculated for the dyspnea ratings of the dyspnea exposure phase which were followed up by dependent *t*-tests. Finally, a dependent *t*-test was calculated for the MIP between T1 and T5. The degrees of freedom were corrected in case of deviance from sphericity using Huynh–Feldt correction (Huynh and Feldt, 1976). Partial eta-squared (η^2^_p_) and *r* were used as a measure of effect size for the ANOVA and dependent *t*-test calculations, respectively (Cohen, 1973; Field, 2009). The level of significance was set to *p* < 0.05. If a significant interaction occurred, we refrained from reporting the main effects in order to avoid erroneous interpretation (Aiken and West, 1991). Instead, simple effect tests were computed as follow-up tests using a hierarchical approach as suggested by Cohen (2008), i.e., a significant three-way interaction was followed up by investigating all three two-way interactions and main effect combinations. A modified version of the false discovery rate (B-Y method) was used to be able to control for multiple testing (Benjamini and Yekutieli, 2001; Narum, 2006).

**Table 1:** Characteristics of the participants.

Males/Females	9/22
Age (years)	21.58 (2.45)
Weight (kg)	66.29 (11.82)
Height (cm)	171.29 (8.23)
Resistive load level (cmH_2_O/l/s)	34.68 (17.03)
Trait version state-trait anxiety inventory score	33.90 (7.80)
State version state-trait anxiety inventory score	37.10 (8.81)


*Data are presented as n or mean (*SD*).*

Sensitivity analysis of the repeated measure ANOVAs using G^∗^Power 3.1.9.2 (Erdfelder et al., 1996) showed that the present sample size is sufficient to detect medium effects for the experimental conditions (2 measurements, η^2^ = 0.063) and medium effects for the dyspnea exposure phase (5 measurements, η^2^ = 0.039) with α = 0.05 and power (1 – β) = 0.80.

Anxiety levels were investigated by correlating STAI scores with the reaction times, accuracy, LPC, N400, ratings (dyspnea, affective), and respiratory variables using Spearman rank correlation coefficients (*r_s_*). In order to keep the result section concise solely significant correlations were reported.

## Results

### Ratings of Dyspnea During Experimental Conditions

Table 2 shows the dyspnea ratings for both experimental conditions on T1 and T5. Two separate 2 (condition) x 2 (time) ANOVAs were calculated for dyspnea intensity and dyspnea unpleasantness. A significant interaction effect between condition and time for dyspnea intensity ratings was found, *F*(1,30) = 5.08, *p* = 0.032, η^2^_p_ = 0.15. Simple effect tests (B-Y corrected for four comparisons: *p* < 0.024) indicated increased dyspnea intensity ratings for the dyspnea compared to the baseline condition for T1 and T5 indicating a successful dyspnea induction (both *p*s < 0.001, Table 2). Furthermore, solely during the dyspnea condition dyspnea intensity was reduced during T5 compared to T1 (*p* = 0.010, Table 2) while ratings for the baseline condition were comparable between T5 and T1 (*p* > 0.26).

**Table 2:** Means (*SD*) for ratings of dyspnea and affective state for each condition for T1 and T5.

	T1		T5	
		
	Baseline	Dyspnea	Baseline	Dyspnea
Dyspnea intensity (VAS)	8.55 (10.66)	45.61 (21.75)	6.87 (10.21)	37.66 (24.99)
Dyspnea unpleasantness (VAS)	8.27 (12.53)	35.66 (26.10)	5.58 (9.66)	34.37 (27.56)
Valence (SAM)	6.08 (1.57)	5.52 (1.70)	6.27 (1.56)	6.06 (1.56)
Arousal (SAM)	3.58 (1.48)	4.11 (1.64)	3.16 (1.40)	3.42 (1.46)


*VAS, visual analog scale; SAM, Self-Assessment Manikin; T1, first testing day; T5, fifth testing day. Lower valence ratings indicate more unpleasantness.*

Dyspnea unpleasantness ratings differed significantly for the factor condition, *F*(1,30) = 41.97, *p* < 0.001, η^2^_p_ = 0.58; but were comparable for the factor time, *F*(1,30) = 0.81, *p* = 0.38, η^2^_p_ = 0.03, with lower dyspnea unpleasantness ratings for the baseline compared to the dyspnea condition indicating a successful dyspnea induction (Table 2). No significant interaction effect was found, *F*(1,30) = 0.19, *p* = 0.67, η^2^_p_ = 0.01.

No significant correlations were found between the abovementioned ratings and the STAI-S and STAI-T ratings with the highest correlation being, *r_s_* = 0.33, *p* = 0.07.

### Ratings of Affective State During Experimental Conditions

Table 2 shows ratings for valence and arousal for both experimental conditions on T1 and T5. Two separate 2 (condition) × 2 (time) ANOVAs were calculated for both affective ratings. Valence ratings were significantly different for the factor condition, *F*(1,30) = 9.62, *p* = 0.004, η^2^_p_ = 0.24; and for the factor time, *F*(1,30) = 4.26, *p* = 0.048, η^2^_p_ = 0.12, with higher valence ratings (=more pleasantness) for baseline compared to dyspnea condition as well as higher valence ratings during T5 compared to T1 (Table 2). No significant interaction effect was found, *F*(1,30) = 3.59, *p* = 0.07, η^2^_p_ = 0.11.

Arousal ratings differed significantly for the factor condition, *F*(1,30) = 12.80, *p* = 0.001, η^2^_p_ = 0.30; and for the factor time, *F*(1,30) = 10.93, *p* = 0.002, η^2^_p_ = 0.27, with lower arousal ratings for the baseline compared to dyspnea condition as well as generally lower arousal ratings during T5 compared to T1 (Table 2). No significant interaction effect was observed, *F*(1,30) = 2.65, *p* = 0.11, η^2^_p_ = 0.08.

STAI-S ratings were significantly negatively correlated with all valence ratings (all *p*s < 0.001; T1 valence baseline: *r_s_* = -0.57; T1 valence dyspnea: *r_s_* = -0.61; T5 valence baseline: *r_s_* = -0.68; T5 valence dyspnea: *r_s_* = -0.62). Additionally, STAI-T ratings were solely significantly negatively correlated with both valence ratings from T5 (all *p*s < 0.020; T5 valence baseline: *r_s_* = -0.42; T5 valence dyspnea: *r_s_* = -0.48). Arousal ratings showed no significant correlations with the highest correlation being, *r_s_* = 0.27, *p* = 0.14.

### Ratings of Dyspnea During Dyspnea Exposure Phases

**FIGURE 2:**
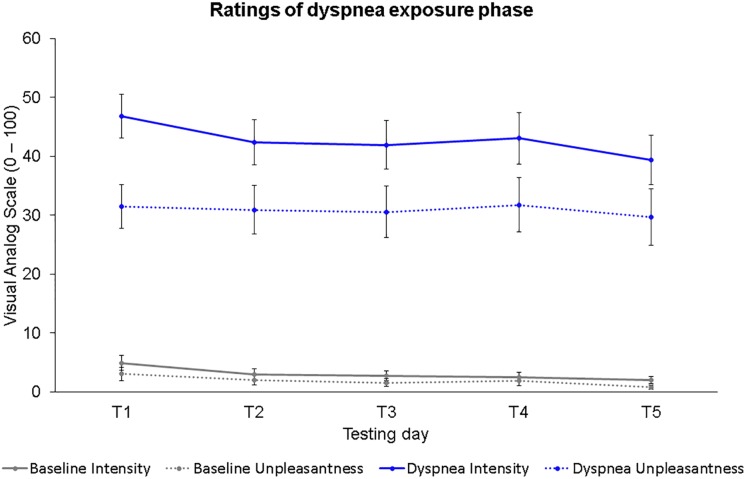
Dyspnea intensity and dyspnea unpleasantness ratings for all five testing days during the dyspnea exposure phases. Dots represent mean values. Error bars represent standard errors.

Figure 2 shows the dyspnea intensity and dyspnea unpleasantness ratings for each dyspnea exposure phase on all five testing days (T1–T5). Two separate 2 (condition) × 5 (time) ANOVAs were calculated for dyspnea intensity and dyspnea unpleasantness ratings. Dyspnea intensity ratings differed significantly for the factor condition, *F*(1,30) = 121.34, *p* < 0.001, η^2^_p_ = 0.80; and for the factor time, *F*(4,120) = 4.40, *p* = 0.002, η^2^_p_ = 0.13, with lower dyspnea intensity ratings for baseline compared to the dyspnea condition confirming a successful dyspnea induction as well as lower dyspnea intensity ratings with increasing time. No significant interaction effect was found, *F*(4,120) = 0.92, *p* = 0.45, η^2^_p_ = 0.03. Most importantly, we found a significant (B-Y corrected for ten comparisons: *p* < 0.017) reduction in dyspnea intensity ratings between T1 and T5 for the baseline, *t*(30) = 3.19, *p* = 0.003, *r* = 0.50; and dyspnea condition, *t*(30) = 2.76, *p* = 0.010, *r* = 0.45.

Dyspnea unpleasantness ratings were significantly different for the factor condition, *F*(1,30) = 55.66, *p* < 0.001, η^2^_p_ = 0.65; but not for the factor time, *F*(3.29,98.77) = 0.65, *p* = 0.60, η^2^_p_ = 0.02, with lower dyspnea unpleasantness ratings for baseline compared to the dyspnea condition. No significant interaction effect was observed, *F*(3.19,95.58) = 0.09, *p* = 0.97, η^2^_p_ = 0.00.

Dyspnea intensity and dyspnea unpleasantness ratings were not significantly correlated with STAI-S and STAI-T scores with the highest correlation being, *r_s_* = 0.30, *p* = 0.10.

### Respiratory Variables During the Experimental Conditions

We calculated six separate 2 (condition) × 2 (time) ANOVAS for the respiratory variables including f, T_I_, V_T_, V’, P_Imax_, and PETCO_2_ (B-Y corrected for six comparisons: *p* < 0.020). Respiratory variables for both experimental conditions as well as T1 and T5 are presented in Table 3.

**Table 3:** Means (*SD*) of respiratory variables for each condition for T1 and T5.

	T1		T5	
		
	Baseline	Dyspnea	Baseline	Dyspnea
Breathing frequency (f), breaths/minute	15.46 (2.88)	12.84 (3.92)	14.78 (3.49)	12.32 (3.16)
Inspiratory time (T_I_), s	1.71 (0.32)	3.26 (1.85)	1.83 (0.36)	3.37 (1.81)
Tidal volume (V_T_), l	0.59 (0.11)	0.67 (0.20)	0.57 (0.09)	0.64 (0.14)
Mean airflow (V’), l/s	0.35 (0.07)	0.23 (0.06)	0.32 (0.06)	0.22 (0.05)
Peak inspiratory mouth pressure (P_Imax_), cmH_2_O	-0.49 (2.80)	-10.31 (4.54)	-0.77 (2.09)	-10.47 (4.73)
End-tidal CO_2_ (PETCO_2_), %	35.15 (3.76)	37.11 (3.94)	34.50 (3.92)	36.11 (3.70)


*T1, first testing day; T5, fifth testing day.*

Significant differences for f, T_I_, V_T_, P_Imax_, PETCO_2_ were found for the factor condition (*p* < 0.001), but not for the factor time (*p* > 0.08), with higher f and lower T_I_,V_T_, P_Imax_, PETCO_2_ for the baseline compared to the dyspnea condition (Table 3). No significant interaction effects were found for f, T_I_, V_T_, P_Imax_, PETCO_2_ (all *p*s > 0.18).

A significant interaction effect between condition and time for V’ was found, *F*(1,30) = 7.74, *p* = 0.009, η^2^_p_ = 0.21. Simple effect tests (B-Y corrected for four comparisons: *p* < 0.024) demonstrated significantly reduced V’ for T5 compared to T1 for the baseline (*p* = 0.003) but not for the dyspnea condition (*p* = 0.31). Furthermore, V’ was higher during baseline compared to the dyspnea condition on T1 and T5 (both *p*s < 0.001, Table 3).

Additionally, we calculated a dependent *t*-test for the MIP to compare inspiratory muscle strength between T1 (*M* = 82.25, *SD* = 21.11) and T5 (*M* = 84.96, *SD* = 21.74). We found no significant difference between both testing days, *t*(23)^1^ = -1.48, *p* = 0.15, *r* = 0.29.

STAI-S ratings were not significantly correlated with any respiratory variable, while STAI-T ratings yielded only one significant correlation with V_T_ during the baseline condition for T1, *r_s_* = 0.39, *p* = 0.031.

### Behavioral Performance

Two separate 2 (condition) × 2 (congruency) × 2 (time) ANOVAs were calculated for behavioral measurements including reaction times and accuracy. Reaction times did not differ significantly for the factor condition, *F*(1,30) = 1.73, *p* = 0.19, η^2^_p_ = 0.05; but for the factor congruency, *F*(1,30) = 148.22, *p* < 0.001, η^2^_p_ = 0.83; and time, *F*(1,30) = 23.17, *p* < 0.001, η^2^_p_ = 0.44. Incongruent color-words elicited prolonged reaction times compared to congruent color-words for both testing days. Furthermore, general faster reaction times were observed during T5 compared to T1 indicating training effects similar to those reported by Strauss (2005, Table 4). No significant two- or three-way interactions were observed (*p* > 0.11).

**Table 4:** Means (*SD*) of behavioral measures for each condition for T1 and T5.

	T1		T5	
		
	Baseline	Dyspnea	Baseline	Dyspnea
Reaction time_congruent_ (ms)	724.03 (124.03)	739.48 (143.96)	666.57 (112.56)	676.09 (116.45)
Reaction time_incongruent_ (ms)	815.29 (124.26)	823.26 (153.63)	741.50 (120.27)	755.45 (138.04)
Accuracy_congruent_ (errors in %)	5.16 (4.50)	4.51 (2.92)	3.68 (2.64)	3.31 (3.60)
Accuracy_incongruent_ (errors in %)	5.53 (3.95)	6.45 (4.22)	4.08 (3.23)	4.48 (4.54%)


*T1, first testing day; T5, fifth testing day.*

Accuracy did not differ significantly for the factor condition, *F*(1,30) = 0.06, *p* = 0.82, η^2^_p_ = 0.00; but for the factor congruency, *F*(1,30) = 7.21, *p* = 0.012, η^2^_p_ = 0.19; and time, *F*(1,30) = 8.32, *p* = 0.007, η^2^_p_ = 0.22. Generally, less accuracy was found for incongruent compared to congruent color-words as well as increased accuracy during T5 compared to T1 indicating training effects (Table 4). No significant two- or three-way interactions were observed (*p* > 0.13).

Furthermore, we examined whether the accuracy differences reported in Sucec et al. (2018b) were also evident in this sample. Here, we calculated the same tests as reported in Sucec et al. (2018b). Similarly, accuracy (error rate in percent) during T1 was lower during the dyspnea condition for incongruent color-words (*M* = 6.45%, *SD* = 4.22%) as compared to congruent color-words (*M* = 4.51%, *SD* = 2.92%), *t*(30) = -3.33, *p* = 0.002, *r* = 0.52 but not during the baseline condition (*p* = 0.65). There were no differences between both conditions for incongruent and congruent color-words during T5 (both *p*s > 0.22; Table 4).

STAI-S and STAI-T ratings were not significantly correlated with any variable of behavioral performance with the highest correlation being, *r_s_* = -0.33, *p* = 0.07.

### Late Positive Complex

**FIGURE 3:**
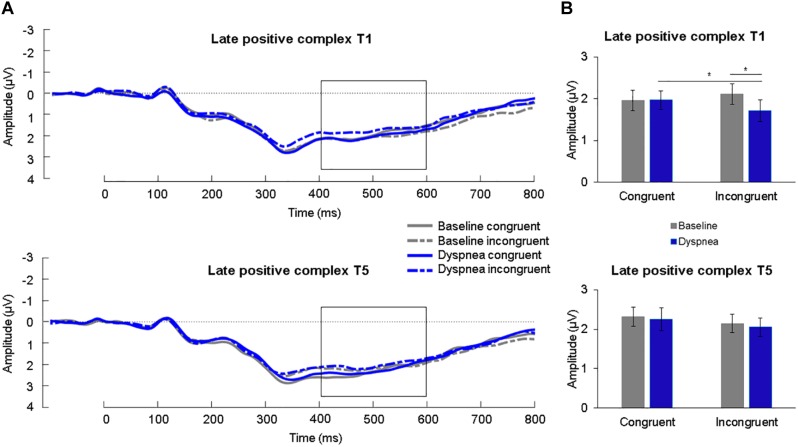
**(A)** Grand average waveforms (μV) for each condition at centro-parietal sites for congruent and incongruent stimuli for T1 (upper panel) and T5 (lower panel). The late positive complex (LPC) is highlighted from 400 to 600 ms. **(B)** Mean (*SE*) amplitudes (μV) for each condition for the LPC for congruent and incongruent stimuli for T1 (upper panel) and T5 (lower panel). ^∗^*p* < 0.05.

**FIGURE 4:**
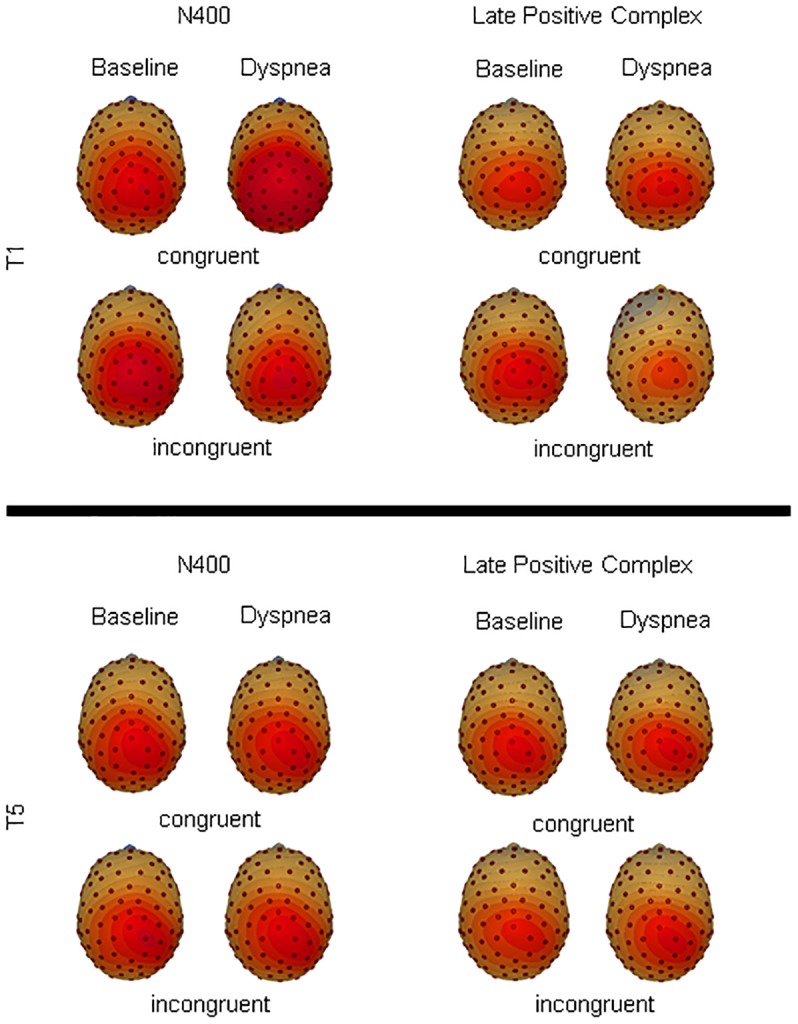
Topographical illustration of the late positive complex and N400 for congruent and incongruent color-words during each condition for T1 and T5.

A 2 (condition) × 2 (congruency) × 2 (time) ANOVA for the LPC was calculated. A significant three-way interaction was found, *F*(1,30) = 6.74, *p* = 0.014, η^2^_p_ = 0.18. Simple effects tests (B-Y corrected for six comparisons: *p* < 0.020) revealed a significant simple interaction effect between condition and congruency for T1 (*p* = 0.002*)* but not for T5 (*p* = 0.87). The significant simple interaction effect was further followed up (B-Y corrected for four comparisons: *p* < 0.024) and revealed for T1 a reduced LPC for incongruent (*M* = 1.71 μV, *SD* = 1.42 μV) compared to congruent (*M* = 1.97 μV, *SD* = 1.25 μV) color-words during the dyspnea condition (*p* = 0.020) as well as a reduced LPC during dyspnea (*M* = 1.71 μV, *SD* = 1.42 μV) compared to the baseline condition (*M* = 2.11 μV, *SD* = 1.37 μV) during incongruent color-word presentation (*p* = 0.015; Figure 3, 4). All other comparisons were non-significant. Overall, these results are highly similar to the findings reported in Sucec et al. (2018b) where a similarly decreased LPC during dyspnea for incongruent compared to congruent color-words for T1 were found.

STAI-S and STAI-T ratings were not significantly correlated with the LPC with the highest correlation being, *r_s_* = -0.17, *p* = 0.37.

### N400

**FIGURE 5:**
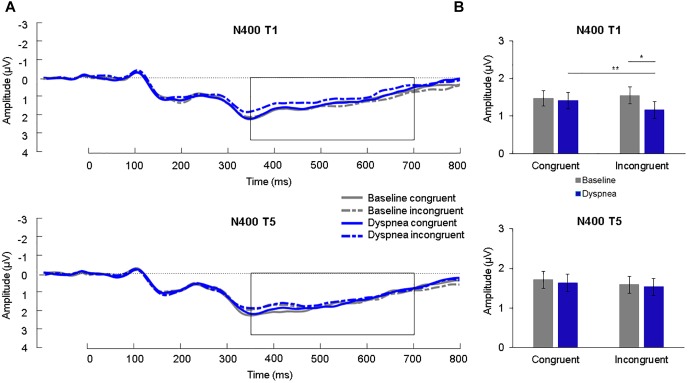
**(A)** Grand average waveforms (μV) during each condition at centro-parietal sites for congruent and incongruent stimuli for T1 (upper panel) and T5 (lower panel). The N400 is highlighted from 350 to 700 ms. **(B)** Mean (*SE*) amplitudes (μV) during each condition for the N400 for congruent and incongruent stimuli for T1 (upper panel) and T5 (lower panel). ^∗^*p* < 0.05, ^∗∗^*p* < 0.01.

A 2 (condition) × 2 (congruency) × 2 (time) ANOVA for the N400 was calculated. The N400 showed a significant three-way interaction, *F*(1,30) = 8.89, *p* = 0.006, η^2^_p_ = 0.23. Simple effects tests (B-Y corrected for six comparisons: *p* < 0.020) showed a significant simple interaction effect between condition and congruency for T1 (*p* = 0.001*)* but not for T5 (*p* = 0.83). The significant simple interaction effect of T1 was further followed up (B-Y corrected for four comparisons: *p* < 0.024) and revealed an increased (=more negative) N400 for dyspnea (*M* = 1.16 μV, *SD* = 1.27 μV) compared to the baseline condition (*M* = 1.55 μV, *SD* = 1.24 μV) for the incongruent color-words (*p* = 0.002) as well as an increased N400 for incongruent color-words (*M* = 1.16 μV, *SD* = 1.27 μV) compared to congruent words (*M* = 1.42 μV, *SD* = 1.19 μV) during the dyspnea condition (*p* = 0.011; Figure 5). Overall, these results are highly similar to the findings by Sucec et al. (2018b) who reported increased N400 mean amplitudes for incongruent stimuli during dyspnea as compared to baseline for T1.

STAI-S and STAI-T ratings were not significantly correlated with N400 mean amplitudes with the highest correlation being, *r_s_* = 0.22, *p* = 0.24.

## Discussion

Previous literature demonstrated an impairing effect of dyspnea on RI in healthy individuals (Sucec et al., 2018b). However, the effect of repeated dyspnea exposure on RI has not been examined. Therefore, the aim of the current study in healthy participants was to investigate the effects of repeated dyspnea exposure over a 5-day period on RI. Since previous literature showed habituation effects after repeated induction of dyspnea (Wan et al., 2009; Stoeckel et al., 2015), it was hypothesized that repeated dyspnea exposure over 5 days would cause dyspnea habituation resulting in a reduction of the impairing effect of dyspnea on RI. Particularly, less impairment of dyspnea on RI as indicated by more comparable behavioral measures (reaction times and accuracy) and neural processing (LPC and N400) between the baseline and dyspnea condition during T5 relative to T1 was predicted. To the best of our knowledge, such effects of repeated dyspnea exposure on RI over a longer time period than one testing day have not been investigated before.

The induction of dyspnea was confirmed in the dyspnea condition for all testing days by ratings of dyspnea intensity and dyspnea unpleasantness, and respiratory variables, which is in line with previous studies using comparable dyspnea induction methods (von Leupoldt et al., 2008; Alexander-Miller and Davenport, 2010; Benke et al., 2017; Juravle et al., 2017; Herzog et al., 2018a; Sucec et al., 2018a).

As expected, participants showed habituation of dyspnea after undergoing the dyspnea exposure phases for five testing days. This was indicated by reduced dyspnea intensity ratings of the dyspnea exposure phase during T5 compared to T1 as well as by reduced dyspnea intensity and arousal ratings, and increased valence ratings during T5 compared to T1 during the Stroop task. The habituation of dyspnea over the five testing days was further supported by comparable MIP values for T1 and T5 which suggests that this habituation effect did not occur due to an inspiratory muscle training effect. Furthermore, respiratory variables during dyspnea induction were comparable between T1 and T5, which is an important indication that the observed habituation effects cannot be explained by any adaptions or changes in breathing patterns over time. Somewhat surprisingly, dyspnea unpleasantness was comparable between T1 and T5 suggesting that habituation occurred only in the sensory (intensity) but not in the affective dimension (unpleasantness) of dyspnea. This is in contrast to previous studies which typically found habituation of dyspnea intensity and/or unpleasantness (Wan et al., 2009; Stoeckel et al., 2015). However, it is important to note that these studies investigated habituation within one testing day whereas the current study investigated habituation within shorter blocks over five testing days. This suggests that habituation of dyspnea exposure over several testing days might yield different results as dyspnea exposure over only one testing day. This would be in line with a recent study by Hayen et al. (2015) who also found no habituation effects of dyspnea unpleasantness over a four-day exposure period. However, the current study, as well as the study by Hayen et al. (2015), tested exclusively healthy participants in a laboratory setting using resistive load induced dyspnea. Previous studies testing dyspneic patients with COPD over several days in a more clinical setting have demonstrated reductions in dyspnea unpleasantness (Carrieri-Kohlman et al., 2001; Wadell et al., 2013; Donesky et al., 2014; Herigstad et al., 2017). Therefore, it might alternatively be speculated that habituation of dyspnea over several days differs between patients and controls as well as between different test settings, which necessitates future studies directly addressing these differences.

The expected Stroop effect was evident during T1 and T5 as indicated by prolonged reaction times for incongruent compared to congruent stimuli for both experimental conditions suggesting a successful experimental manipulation of RI (Hanslmayr et al., 2008; Badzakova-Trajkov et al., 2009; Larson et al., 2009). Generally, reaction times were faster and responses more accurate during T5 compared to T1, which can be attributed to general training effects comparable to those reported in previous research using the Stroop color-word task (e.g., Strauss, 2005).

For T1 and T5 reaction times were comparable between the baseline and dyspnea condition showing no general impairing effect of experimentally induced dyspnea on reaction times. However, as expected more errors occurred for incongruent compared to congruent stimuli during dyspnea compared to baseline conditions which was only evident for T1 but not for T5. This suggests that repeated dyspnea exposure reduced the impairing effect of dyspnea on RI resulting in improved accuracy, which became comparable to the baseline condition.

Furthermore, our results show a reduction in LPC as well as an increase in negativity of the N400 during dyspnea compared to baseline for the inhibition-relevant incongruent stimuli during T1. As expected, after undergoing repeated dyspnea exposure these effects were no more present during T5. It might be argued that during T1 inspiratory resistive loads increased the load on the inspiratory muscles leading to muscle fatigue, attention shifts, and a possible overload of sensory information (Gorman et al., 1999). This might have resulted in more cognitive demand for the semantic processing indexed as the increased N400 (Kutas and Federmeier, 2011). As a consequence, fewer resources were available for RI indexed as the partly overlapping reduced LPC during T1. Possibly the habituation toward this “overload” of sensory information reduced the demand for additional resources for the processing of the semantic meaning of the color-words (N400), which resulted in more resources available to inhibit automatic or prepotent responses (LPC) during T5. Furthermore, research showed that the LPC might be considered an indicator for manipulations related to attention, possibly reflecting the processing of representations in the working memory (Cuthbert et al., 2000; Schupp et al., 2006; Luck, 2014). It seems that due to habituation of dyspnea, these impairments found for T1 disappeared, resulting in comparable behavioral performance and electrophysiological processing of the color-words during T5. Additionally, task difficulty might be an important factor as the behavioral performance and neural processing of congruent color-words were not affected by dyspnea induction. This might indicate that dyspnea does not necessarily impair all forms of cognitive processing, but rather causes impairments for more demanding cognitive processes such as processing incongruent color-words. This interpretation is in line with a recent study (Sucec et al., 2018a) that showed no impairing effect of dyspnea on behavioral performance and neural processing during the performance of a relatively simple cognitive task (Flanker task). In sum, a possible dyspnea-cognition interference might be related to task difficulty which necessitates the evaluation of graded task difficulty levels on dyspnea-cognition interference during repeated dyspnea exposures in future studies.

Explorative analyses of the current study found no relationships between anxiety levels and ratings for dyspnea intensity and dyspnea unpleasantness as well as the electrophysiological processing. This is in contrast to Wan et al. (2006, 2012) who found less habituation or even sensitization for repeated dyspnea exposure in high compared to low anxious healthy students and patients. However, contrary to the current study using an ad-hoc sample without preselection based on anxiety levels, Wan et al. (2006) pre-selected their sample based on high vs. low STAI-T levels as well as using a different form of dyspnea induction which might explain the diverging results. Since anxiety is highly prevalent in dyspneic patients (von Leupoldt, 2017) future studies are clearly needed to further investigate the potential effects of anxiety on a possible dyspnea-cognition interference.

Similarly, future studies are necessary to explore how much the current findings translate to clinical dyspneic populations such as asthma and COPD patients. At the current stage, it is unclear whether dyspneic patients might show similar impairments of dyspnea on RI and whether repeated dyspnea exposure might have comparable beneficial effects in these patients. This is important because treatments oftentimes rely heavily on RI and subsequently self-regulation. If repeated dyspnea exposure could positively affect the potentially impairing effect of dyspnea on RI in these patients this might support treatments and facilitate goal-directed behavior.

This study has some limitations that need to be acknowledged. The most important limitation is the absence of a control group. Although unlikely it cannot be completely excluded that habituation effects might have occurred over time in the absence of the dyspnea exposure phase on all five testing days. Moreover, solely healthy participants with rather low anxiety levels took part in the present study, which does not allow a generalization of the results to dyspneic patients or highly anxious individuals. Furthermore, inspiratory resistive loads were the present method of choice to manipulate dyspnea. This method reflects an increase of the dyspnea sensation “work and effort to breathe” which only resembles one dyspnea quality while other qualities (e.g., air hunger) were not studied (Parshall et al., 2012). Thus, future research should apply different dyspnea induction methods including exercise, breathing through collapsible tubes, expiratory resistive loads, CO_2_ inhalation, or even vicarious dyspnea to study their effect on RI over several exposure days (Herzog et al., 2018b).

## Conclusion

The present results demonstrate that the impairing effect of dyspnea on RI found for the first testing day disappeared after 5 days of dyspnea exposure in healthy participants which was evident on a behavioral and electrophysiological level. Future research in dyspneic patient populations is necessary to further substantiate these results.

## Ethics Statement

This study was carried out in accordance with the Declaration of Helsinki and guidelines for good clinical practice (GCP) provided by the medical ethics committee of the hospital of the University of Leuven with written informed consent from all subjects. All subjects gave written informed consent in accordance with the Declaration of Helsinki. The protocol was approved by the medical ethics committee of the hospital of the University of Leuven (S57727).

## Author Contributions

AvL, JS, MH, IV, and OV contributed to conception and design of the study. JS was responsible for data collection and organized the database. JS, MH, and AvL performed the statistical analysis. JS wrote the first draft of the manuscript. JS, MH, OV, IV, and AvL wrote sections of the manuscript. All authors contributed to manuscript revision, read, and approved the submitted version.

## Conflict of Interest Statement

The authors declare that the research was conducted in the absence of any commercial or financial relationships that could be construed as a potential conflict of interest.

## Footnotes

1 Due to device failure the MIP values of seven participants could not be obtained.
